# Supplementary material for: Critical Scaling of Novelty in the Cortex
Source: bioRxiv. 2025 Aug 28:2024.12.23.630084. Preprint. [Version 2] doi: 10.1101/2024.12.23.630084 (PMC12407764; doi:10.1101/2024.12.23.630084)
Supplement: Supplement 1 [file NIHPP2024.12.23.630084v2-supplement-1.pdf]

bioRxiv preprint doi: https://doi.org/10.1101/2024.12.23.630084; this version posted August 28, 2025. The copyright holder for this preprint (which was not certified by peer review) is the author/funder. This article is a US Government work. It is not subject to copyright under 17 USC 105 and is also made available for use under a CC0 license.

Supplementary Figures

Supplementary Figure 1

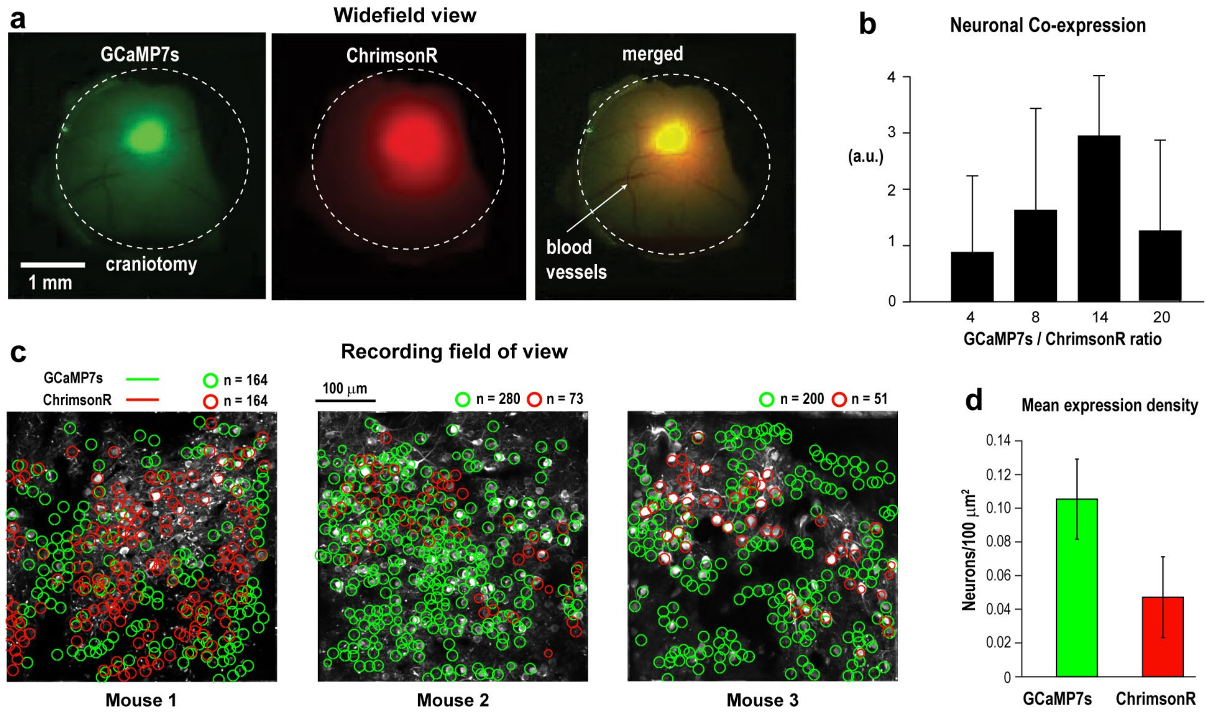

Supplementary Fig. 1 | a Widefield image showing GCaMP7s (*green*) and ChrimsonR (*red*) expression, with the merged image indicating co-expression (single mouse). **b** Optimal co-expression achieved with a virus titer ratio of 14:1 (n = 23 mice). **c** Examples of individual neurons with significant GCaMP7s (*green*) and ChrimsonR (*red*) expression from 3 mice. **d** Mean expression density showing twice as many neurons positive for GCaMP7s compared to ChrimsonR (from c).

bioRxiv preprint doi: https://doi.org/10.1101/2024.12.23.630084; this version posted August 28, 2025. The copyright holder for this preprint (which was not certified by peer review) is the author/funder. This article is a US Government work. It is not subject to copyright under 17 USC 105 and is also made available for use under a CC0 license.

Supplementary Figure 2

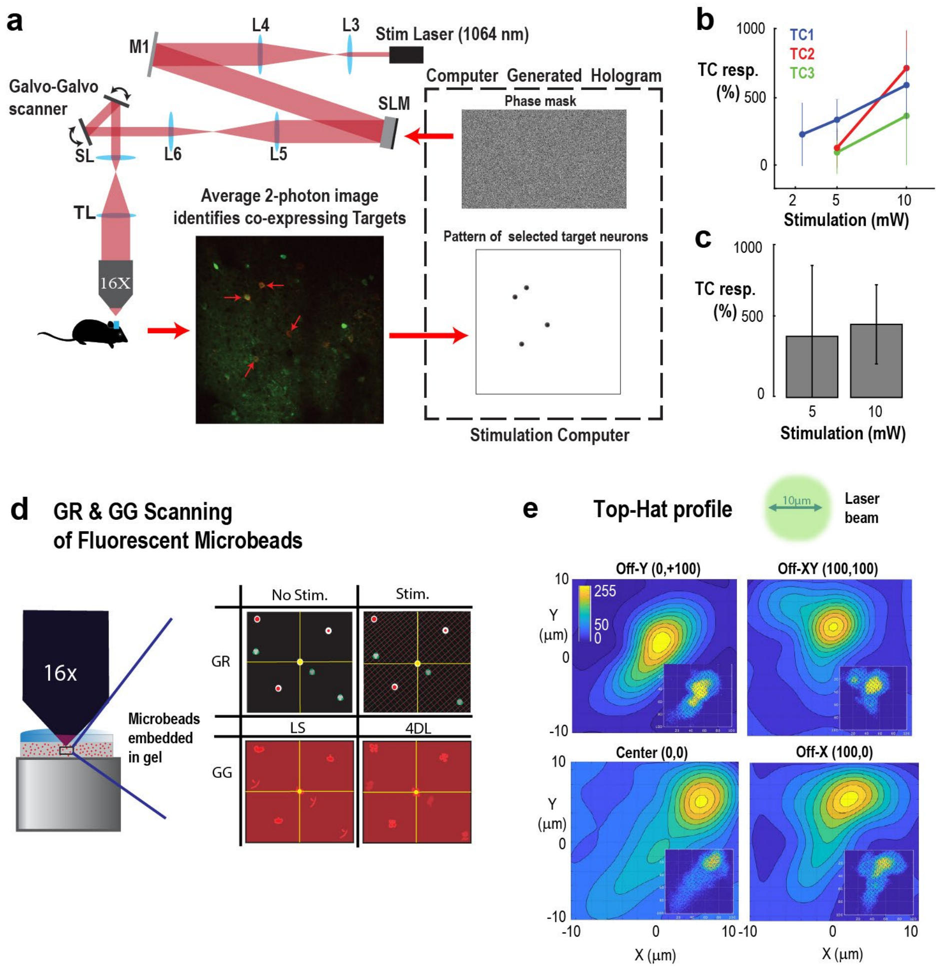

Supplementary Fig. 2 | Schematic design and evaluation of the stimulation path using spatial light modulation. a Laser light (Light Conversion, 1064 nm, 600 kHz, <100 fs) is expanded to fill the area of the spatial light modulator (SLM; Meadowlark, 1920×1152 resolution). The hologram is resized to fit the Galvo-Galvo scanners (~3 mm diameter), which guide the stimulation beam in the focal image plane (Nikon 16X, 3 mm working distance, 12.5 mm effective focal length). Target cells (TC) expressing both GCaMP7s and ChrimsonR (opsin with tdTomato tag) are selected from the mean 2PI image of the field of view from both red and green channels for stimulation and simultaneous estimation of the evoked spike count. Map of TC informs a hologram of top-hat patterns with diameter of 10 µm for the SLM. Optical power was adjusted to 5—10 mW per TC, depending on the depth and expression level. **b** Spiking above base line increases with stimulation power (N = 3 TC; 100 ms stimulation duration). **c** No difference in evoked spike count for 5 and 10 mW stimulation across all TC. **d** Schematics of the stimulation xy-profile reconstruction using fluorescence microbeads. Galvo-Galvo (GG) controlled stimulation of gel-embedded microbeads (3 µm diameter) and simultaneous Galvo-Resonance (GR) 2PI of elicited response. *Circles*: Individual microbeads before and during stimulation. **e** Experimentally reconstructed and smoothed xy-profile of a flat-hat stimulation (10 µm diameter; 5 mW) at 4 different positions within the field of view at center (0,0), off-x (+100 µm, 0), off-y (0, +100 µm), and off-xy (+100 µm, +100 µm). *Inset*: Non-smoothed reconstruction.

bioRxiv preprint doi: https://doi.org/10.1101/2024.12.23.630084; this version posted August 28, 2025. The copyright holder for this preprint (which was not certified by peer review) is the author/funder. This article is a US Government work. It is not subject to copyright under 17 USC 105 and is also made available for use under a CC0 license.

Supplementary Figure 3

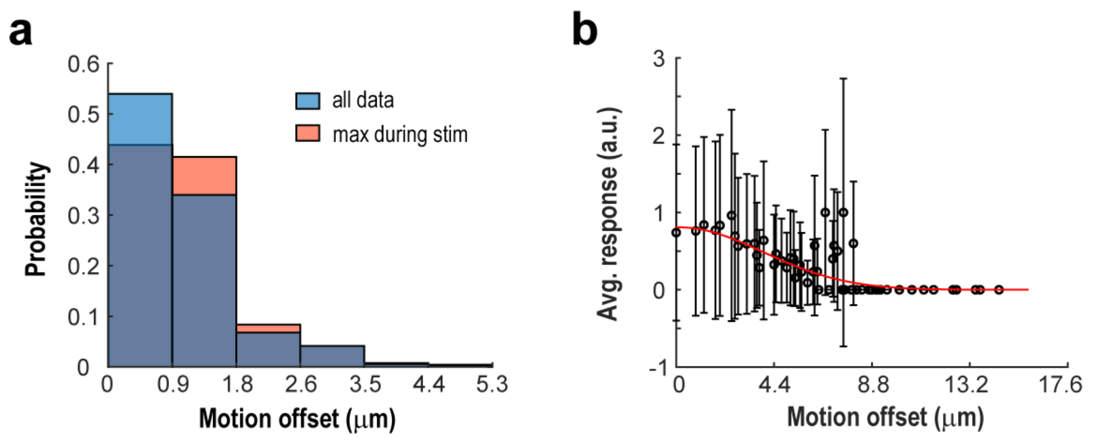

Supplementary Fig. 3 | Analysis of motion correction from processed recordings reveals low amount of motion artifact affecting stimulation. a Distribution of motion offsets observed on each frame over all recordings. *Blue*: all data. *Orange*: maximum displacement observed during stimulation for each trial. **b** Spatial profile of stimulation response from targets measured using motion offset in trials during stimulation. Note that responses drop to approximately zero in frames with an offset above ~4.4 μm.

bioRxiv preprint doi: https://doi.org/10.1101/2024.12.23.630084; this version posted August 28, 2025. The copyright holder for this preprint (which was not certified by peer review) is the author/funder. This article is a US Government work. It is not subject to copyright under 17 USC 105 and is also made available for use under a CC0 license.

Supplementary Figure 4

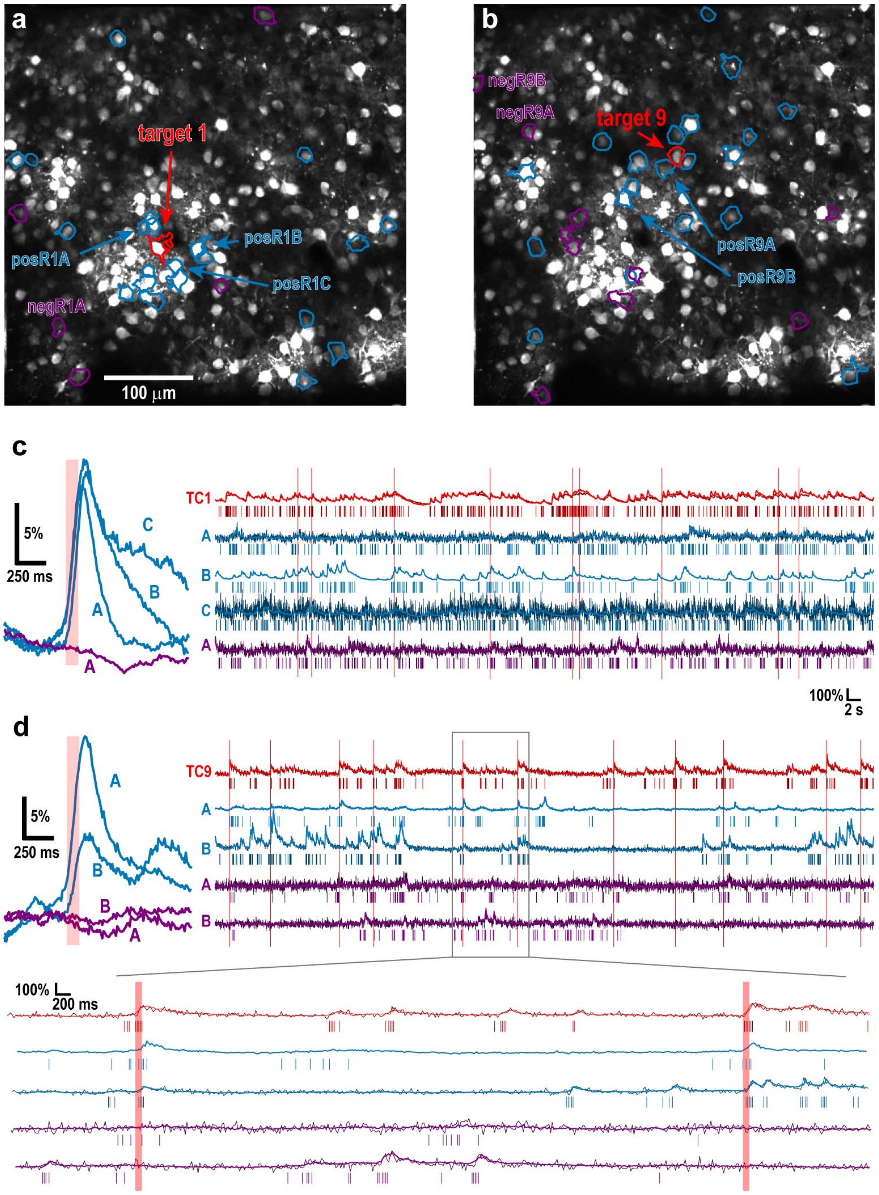

Supplementary Fig. 4 | Example recording with activity from two example targets and some of their significant responders highlighted. a Example field of view with target 1 highlighted in *red* and its positive (*blue*) and negative (*purple*) responders indicated. **b** Same recording as in a, but now highlighting target 9 and its significant responders. **c** *Left*: time average fluorescent response to stimulation for the significant responders labeled in a. *Right*: example raw (*dark*) and denoised (*light*) fluorescent traces (*lines*) and deconvolved spikes (*bars*) for the labeled neurons in a. **d** *Left*: time average fluorescent response to stimulation for the significant responders labeled in b. *Right*: example raw (*dark*) and denoised (*light*) fluorescent traces (*lines*) and deconvolved spikes (*bars*) for the labeled neurons in b. *Bottom*: zoom in from the right panel above.

bioRxiv preprint doi: https://doi.org/10.1101/2024.12.23.630084; this version posted August 28, 2025. The copyright holder for this preprint (which was not certified by peer review) is the author/funder. This article is a US Government work. It is not subject to copyright under 17 USC 105 and is also made available for use under a CC0 license.

Supplementary Figure 5

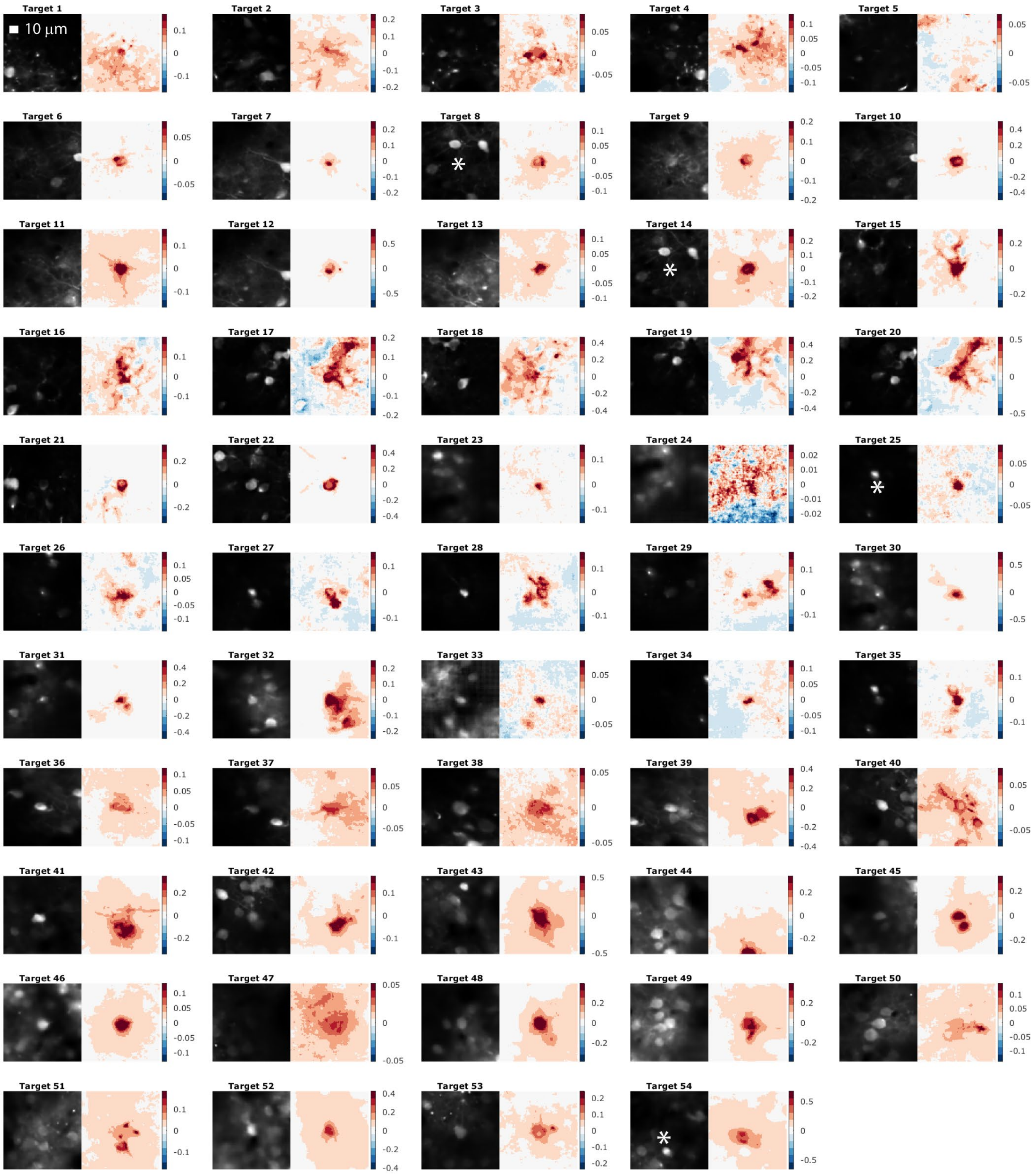

Supplementary Fig. 5 | Summary of isolating individual pyramidal neurons in layer 2/3 of the primary visual cortex during rest using holographic stimulation and 2PI. For each target cell (TC), mean luminance after motion correction and denoising (*left*) and the ΔF/F in response to stimulation in a 100×100-pixel area (~88×88 µm) centered on the TC (*right*) are shown. *Asterisks* are visual guides for TC position.

bioRxiv preprint doi: https://doi.org/10.1101/2024.12.23.630084; this version posted August 28, 2025. The copyright holder for this preprint (which was not certified by peer review) is the author/funder. This article is a US Government work. It is not subject to copyright under 17 USC 105 and is also made available for use under a CC0 license.

Supplementary Figure 6

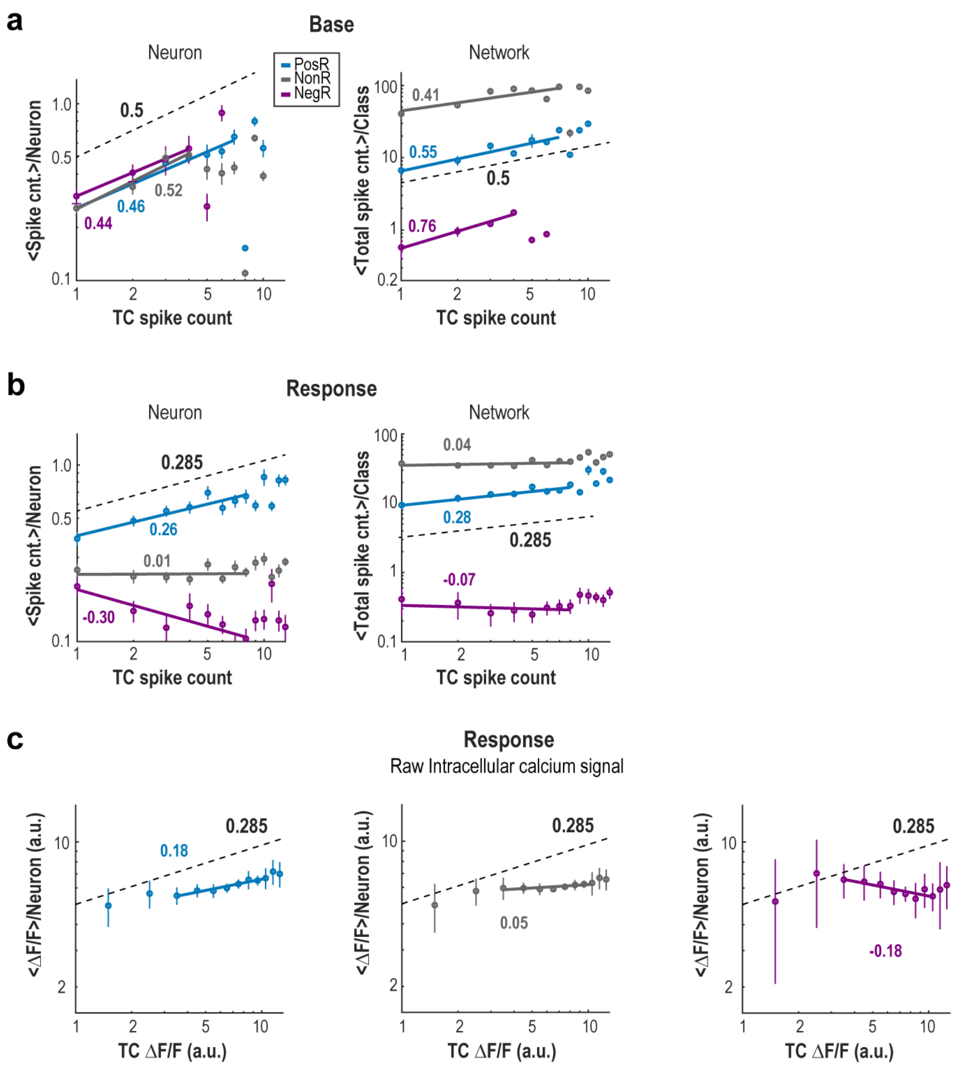

Supplementary Fig. 6 | Spike count in all different subpopulations scale with spike count of target cells during baseline activity, but only Positive Responders scale during stimulation. a Mean spike count on PosR (*blue*), NonR (*grey*) and NegR (*purple*) as function of spike count of TC, in log-log, during baseline. Power law fits are indicated by solid lines, with obtained exponents shown. *Left*: mean calculated per neuron. *Right*: mean calculated after summing over the population. **b** Same as in a, but during holographic stimulation. **c** Response scaling calculated using the fluorescent traces (ΔF/F).

bioRxiv preprint doi: https://doi.org/10.1101/2024.12.23.630084; this version posted August 28, 2025. The copyright holder for this preprint (which was not certified by peer review) is the author/funder. This article is a US Government work. It is not subject to copyright under 17 USC 105 and is also made available for use under a CC0 license.

Supplementary Figure 7

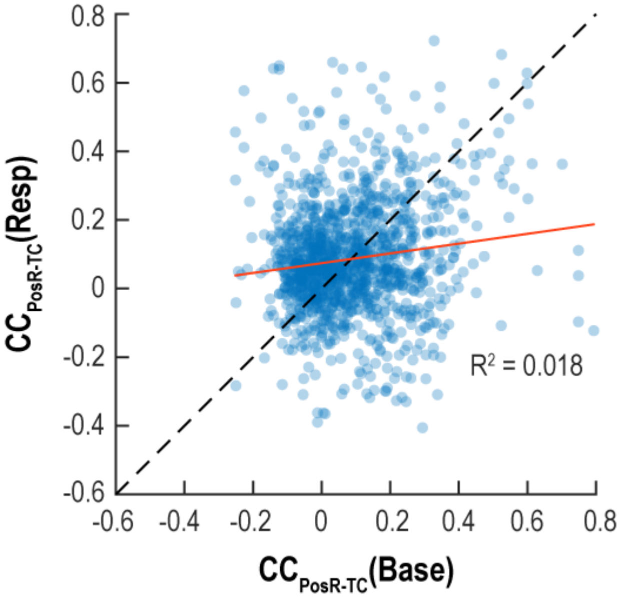

Supplementary Fig. 7 | Correlations between PosR and TC change during stimulation in comparison with baseline. For each PosR-TC pair, their spike count correlation during stimulation is plotted against baseline. Note the shift towards higher values during Resp compared to Base (cp. Fig. 2c). A linear regression (*orange line*) shows that these measures are very weakly related (R^2 = 0.018; linear regression; $p < 10^{-5}$, for the *t*-statistic of the two-sided hypothesis test), indicating that the stimulation of TCs engages both existing but also new networks.

bioRxiv preprint doi: https://doi.org/10.1101/2024.12.23.630084; this version posted August 28, 2025. The copyright holder for this preprint (which was not certified by peer review) is the author/funder. This article is a US Government work. It is not subject to copyright under 17 USC 105 and is also made available for use under a CC0 license.

827 **Supplementary Figure 8**

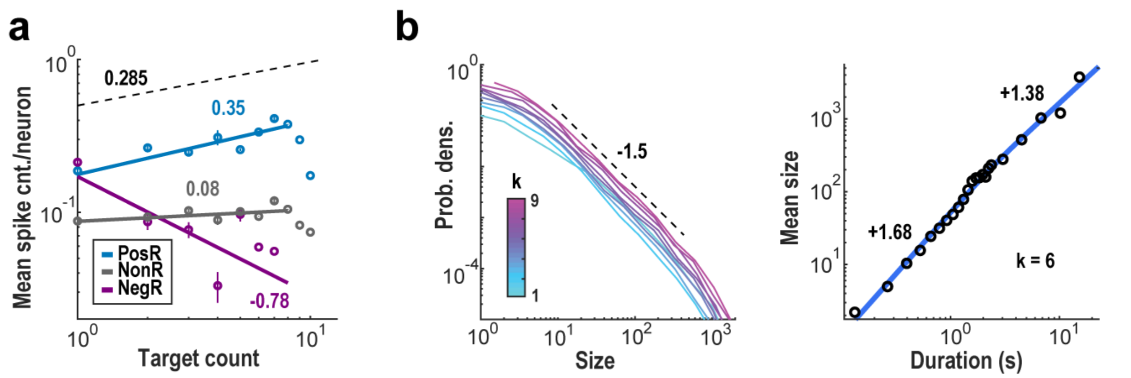

Supplementary Fig. 8 | Response scaling and avalanche analysis are qualitatively unchanged without denoising from Deep Interpolation. a Average response from PosR (*blue*), NonR (*grey*) and NegR (*purple*) as function of TC spike count during stimulation, for datasets processed without denoising (no Deep Interpolation). **b** Avalanche analysis for datasets processed without denoising. *Left*: Distribution of avalanche sizes for different coarse graining levels *k* (*color bar*). *Right*: Mean avalanche size as function of avalanche duration at *k* = 6 showing an exponent below 2 for the short avalanches regime, in line with previous results obtained from noisy datasets (Ribeiro, Capek et al., 2023).

828

bioRxiv preprint doi: https://doi.org/10.1101/2024.12.23.630084; this version posted August 28, 2025. The copyright holder for this preprint (which was not certified by peer review) is the author/funder. This article is a US Government work. It is not subject to copyright under 17 USC 105 and is also made available for use under a CC0 license.

Supplementary Figure 9

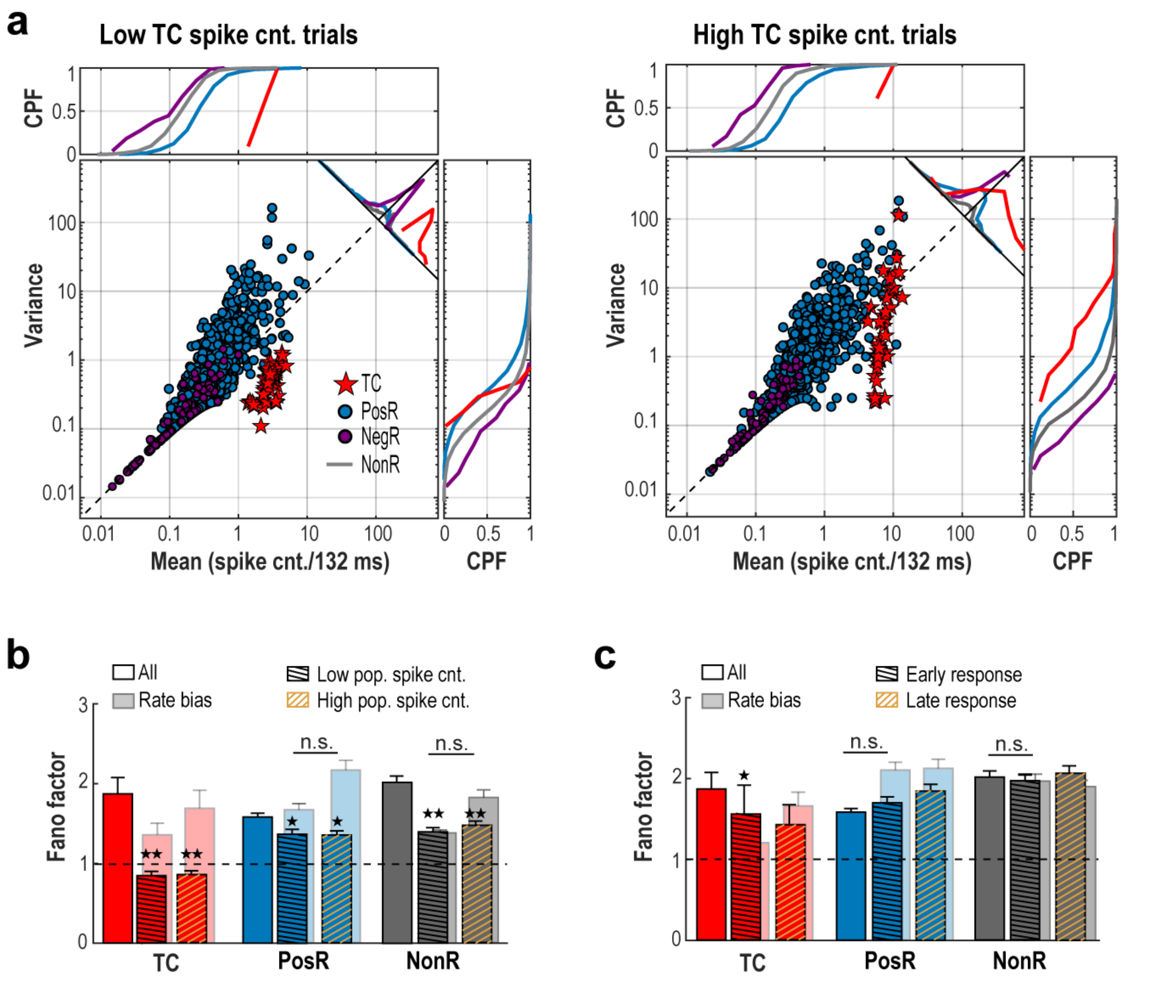

Supplementary Fig. 9 | High response variability in PosR and NonR does not originate from variability in evoked spikes of TC. a Summary statistics of mean TC trials separated by low and high target counts. Note the remaining high variance of PosR despite the large drop in TC variance. No change in Fano Factor (FF) when subdividing into TC trials with high or low preceding population spike count (**b**) or early (first half of the response window) vs. late (second half of the response window) spiking responses (**c**). When comparing FF (b, c), 1 & 2 stars indicate $p < 0.05$ & 10^{-3}, respectively, using Wilcoxon rank sum test.

bioRxiv preprint doi: https://doi.org/10.1101/2024.12.23.630084; this version posted August 28, 2025. The copyright holder for this preprint (which was not certified by peer review) is the author/funder. This article is a US Government work. It is not subject to copyright under 17 USC 105 and is also made available for use under a CC0 license.

Supplementary Figure 10

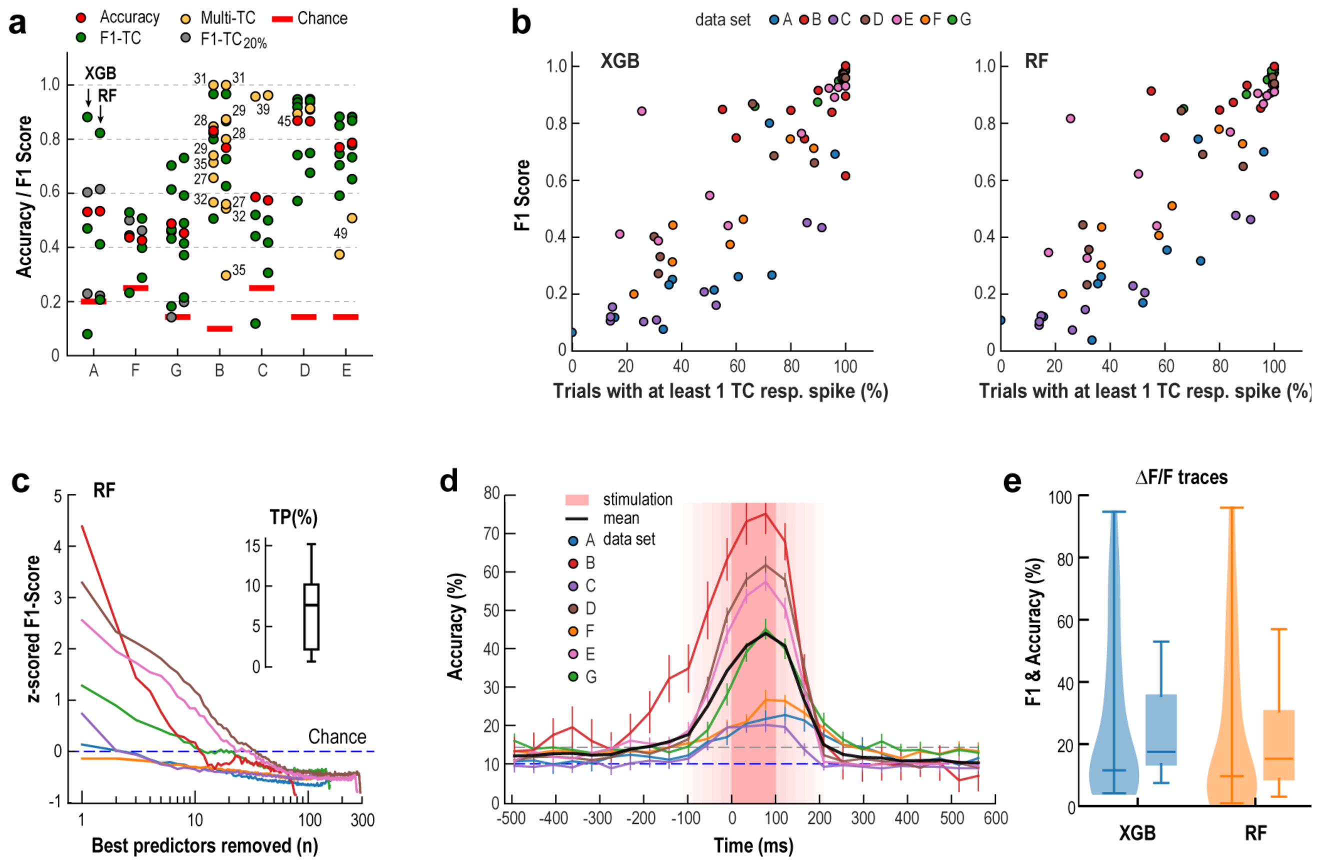

Supplementary Fig. 10 | Decoding accuracy, stimulation efficacy and impact of deep interpolation. a Overview of F1-scores for TCs (*green*) and TCs that did not reach 20% of spike success (*gray*) upon stimulation. F1-scores are compared to chance level (*red bars*) and overall accuracy (*red circles*). XGBoost (XGB; *left*) and Random Forest (RF; *right*) results are very similar across all datasets. Potential multi-cell targets (cp. Supplementary Fig. 5; TCs 27-29, 31-32, 35, 39, 45 and 49) are labeled and marked in *yellow*, and overall do not show above-average decoding success. **b** For each data set, stimulation success (at least 1 spike) varied among TC. Target stimulation success positively correlated with decoding power, i.e. F1-score, of a TC in XGB (*left*) and RF (*right*). **c** Z-scored F1-scores calculated with the Random Forest method for each TC and then averaged, for each dataset (same color code as in Fig. 4), as function of number of best predictors dropped (cp. XGB in Fig. 4c, *right*). *Inset*: Average number of best predictors dropped before F1-scores reach chance levels. **d** Deep Interpolation slightly advances stimulus information and significantly increases accuracy thereby slightly prolonging transient response time. Analysis done over 132-ms window (cp. Fig. 4f, 22-ms window). The enhanced accuracy achieved with Deep Interpolation demonstrates the method's noise-reducing benefits, which improve the model's ability to capture relevant neural signals. However, it also shows that it undermines our ability to study temporal progression of activations, as its window of smoothing ranges across 30 frames. **e** Low (but still above chance) F1 and accuracy when XGB and RF are trained on the raw ΔF/F traces.

bioRxiv preprint doi: https://doi.org/10.1101/2024.12.23.630084; this version posted August 28, 2025. The copyright holder for this preprint (which was not certified by peer review) is the author/funder. This article is a US Government work. It is not subject to copyright under 17 USC 105 and is also made available for use under a CC0 license.

Supplementary Figure 11

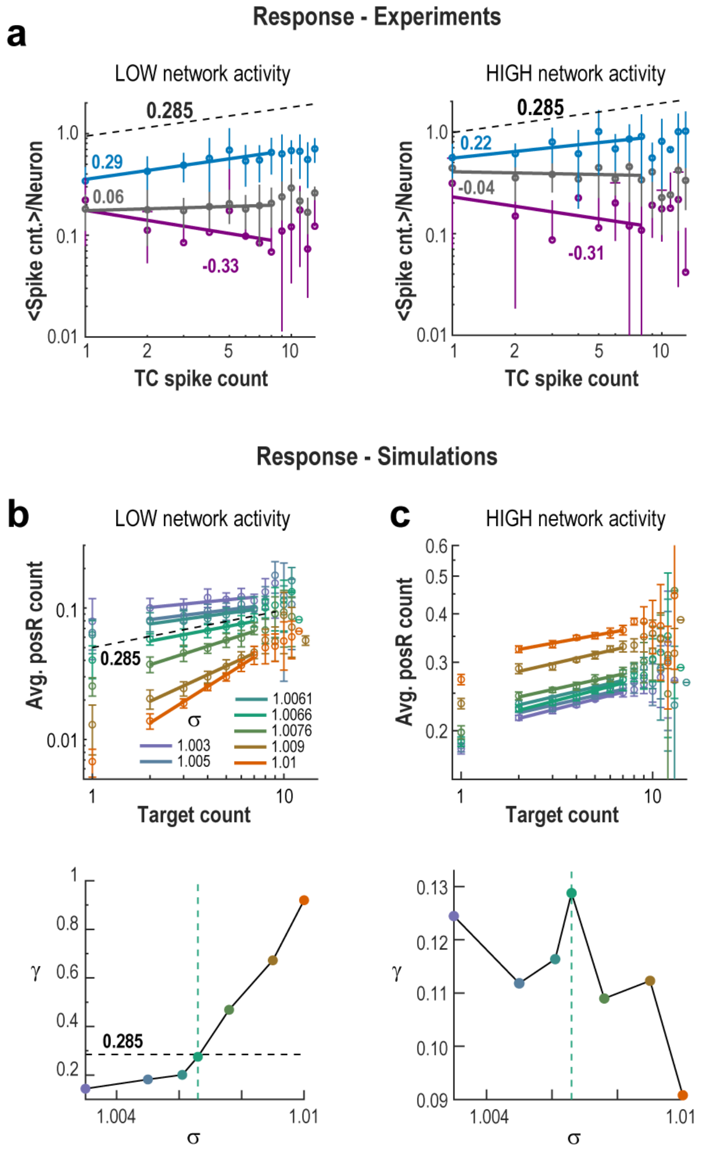

Supplementary Fig. 11 | Spike count in subpopulations scale similar with spike count of target cells during low and high avalanche activity states. a Mean spike count on PosR (*blue*), NonR (*grey*) and NegR (*purple*) as function of spike count of TC, in log-log, during holographic stimulation. Power law fits are indicated by solid lines, with obtained exponents shown. Separating trials with preceding low (*left*) or high (*right*) baseline network activity. Experimental data. **b** Mean response of PosR as function of TC spike count during trials with low base activity for the simulations. *Bottom*: Response scaling exponents as function of branching parameter σ. **c** Same as in b, but for trials with high base activity prior to stimulation.

bioRxiv preprint doi: https://doi.org/10.1101/2024.12.23.630084; this version posted August 28, 2025. The copyright holder for this preprint (which was not certified by peer review) is the author/funder. This article is a US Government work. It is not subject to copyright under 17 USC 105 and is also made available for use under a CC0 license.

836 **Supplementary Figure 12**

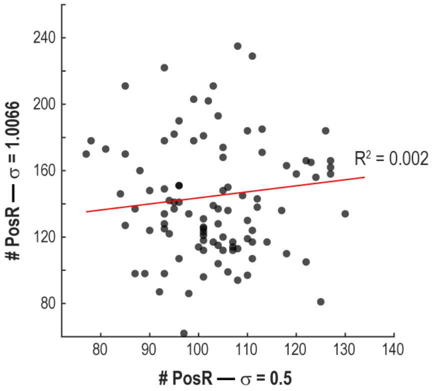

Supplementary Fig. 12 | No correlation between number of PosR for TCs between sub- and critical networks with identical connection structure. Number of PosR for each TC (*circles*) at the critical network (σ = 1.0066) as function of the same measure from a subcritical network (σ = 0.5). From the *t*-statistics of the two-sided hypothesis test we obtained p = 0.27 for the slope of the linear regression (*red line*), with R^2 = 0.002.

837

bioRxiv preprint doi: https://doi.org/10.1101/2024.12.23.630084; this version posted August 28, 2025. The copyright holder for this preprint (which was not certified by peer review) is the author/funder. This article is a US Government work. It is not subject to copyright under 17 USC 105 and is also made available for use under a CC0 license.

Supplementary Table 1

Experimental response scaling exponents (± 95% C.I.)				
**Condition**	**Figure**	**PosR**	**NonR**	**NegR**
Base (neuron)	S6a *left*	0.46 ± 0.07	0.52 ± 0.27	0.44 ± 0.02
Base (network)	2d, S6a *right*	0.55 ± 0.22	0.41 ± 0.27	0.76 ± 0.24
Resp (neuron)	2e, S6b *left*	0.26 ± 0.09	0.01 ± 0.10	-0.30 ± 0.18
Resp (network)	2f, S6b *right*	0.28 ± 0.10	0.04 ± 0.09	-0.07 ± 0.26
Calcium	S6c	0.18 ± 0.04	0.05 ± 0.04	-0.18 ± 0.10
Low	S9 *left*	0.29 ± 0.14	0.06 ± 0.07	-0.33 ± 0.50
High	S9 *right*	0.22 ± 0.21	-0.04 ± 0.18	-0.31 ± 0.66
No DeepIP	S10a	0.35 ± 0.18	0.08 ± 0.09	-0.78 ± 1.48

Supplementary Table 2

Simulation response scaling exponents (± 95% C.I.)			
**Branching parameter**	**Response (Fig. 7b)**	**Low (Fig. 7c, left)**	**High (Fig. 7c, right)**
0.3	0.015 ± 0.048	N/A	N/A
0.5	0.045 ± 0.031	N/A	N/A
0.9	0.105 ± 0.038	N/A	N/A
0.95	0.133 ± 0.040	N/A	N/A
1	0.176 ± 0.112	N/A	N/A
1.003	0.179 ± 0.087	0.144 ± 0.150	0.124 ± 0.015
1.005	0.166 ± 0.044	0.182 ± 0.127	0.112 ± 0.043
1.0061	0.174 ± 0.032	0.201 ± 0.115	0.116 ± 0.019
1.0066	0.247 ± 0.086	0.275 ± 0.080	0.129 ± 0.039
1.0076	0.186 ± 0.082	0.469 ± 0.111	0.109 ± 0.028
1.009	0.198 ± 0.070	0.672 ± 0.108	0.112 ± 0.038
1.01	0.189 ± 0.072	0.920 ± 0.098	0.091 ± 0.013
